# Lysophosphatidylcholine Offsets the Protective Effects of Bone Marrow Mesenchymal Stem Cells on Inflammatory Response and Oxidative Stress Injury of Retinal Endothelial Cells via TLR4/NF-*κ*B Signaling

**DOI:** 10.1155/2021/2389029

**Published:** 2021-10-14

**Authors:** Haijun Zhao, Yanhui He

**Affiliations:** ^1^Department of Pain, The First Hospital of Jilin University, Changchun, 130021 Jilin, China; ^2^Department of Ophthalmology, The Second Hospital of Jilin University, Changchun, 130041 Jilin, China

## Abstract

Diabetic retinopathy (DR), as a major cause of blindness worldwide, is one common complication of diabetes mellitus. Inflammatory response and oxidative stress injury of endothelial cells play significant roles in the pathogenesis of DR. The study is aimed at investigating the effects of lysophosphatidylcholine (LPC) on the dysfunction of high glucose- (HG-) treated human retinal microvascular endothelial cells (HRMECs) after being cocultured with bone marrow mesenchymal stem cells (BMSCs) and the underlying regulatory mechanism. Coculture of BMSCs and HRMECs was performed in transwell chambers. The activities of antioxidant-related enzymes and molecules of oxidative stress injury and the contents of inflammatory cytokines were measured by ELISA. Flow cytometry analyzed the apoptosis of treated HRMECs. HRMECs were further treated with 10-50 *μ*g/ml LPC to investigate the effect of LPC on the dysfunction of HRMECs. Western blotting was conducted to evaluate levels of TLR4 and p-NF-*κ*B proteins. We found that BMSCs alleviated HG-induced inflammatory response and oxidative stress injury of HRMECs. Importantly, LPC offsets the protective effects of BMSCs on inflammatory response and oxidative stress injury of HRMECs. Furthermore, LPC upregulated the protein levels of TLR4 and p-NF-*κ*B, activating the TLR4/NF-*κ*B signaling pathway. Overall, our study demonstrated that LPC offsets the protective effects of BMSCs on inflammatory response and oxidative stress injury of HRMECs via TLR4/NF-*κ*B signaling.

## 1. Introduction

Diabetic retinopathy (DR), as one common complication of diabetes mellitus, is a major cause of blindness worldwide [1]. The severity of DR and the degree of vision loss are associated with the control of blood glucose levels and the length of time for diabetes [2]. Rapid increase of blood glucose in patients with diabetes results in the dysfunction of human retinal microvascular endothelial cells (HRMECs), which is specifically embodied as inflammatory response and oxidative stress injury [3, 4]. Then, retinal capillaries are impaired, capillary endothelial cells begin to proliferate, and the hypoxic omentum tissue releases vascular proliferation substances, which promote the formation of new blood vessels, in turn leading to proliferative diabetic retinopathy (PDR) [5, 6]. Increased inflammation and oxidative stress are identified as key factors in the pathogenesis of DR [7]. Therefore, it is urgently required to elucidate the regulation of high glucose- (HG-) induced oxidative stress and inflammation in HRMECs. The conduction of more comprehensive and logical research is also required to provide more curative options for DR patients.

As we know, pathological retinal neovascularization is the main cause of DR [8]. Antivascular endothelial growth factor (VEGF) therapy has made a breakthrough in retinal neovascularization treatment [9]. However, anti-VEGF therapy was demonstrated to have some side effects, remaining controversial in several aspects [10]. Therefore, it is imperative to develop novel therapeutic strategies against retinal neovascularization. Currently, stem cell therapy has shown satisfied efficacy in DR preclinical models [11]. Among the various stem cells, mesenchymal stem cells, especially those derived from bone marrow, have been explored as a possible treatment for DR [12]. It was previously indicated that bone marrow mesenchymal stem cells (BMSCs) were able to migrate and integrate into the host retina, significantly inhibiting retinal neovascular tufts and remodeling the capillary network [13]. BMSCs secrete paracrine factors to promote vascular regeneration [13]. Herein, we investigated the detailed functions of BMSCs to alleviate DR development by affecting the dysfunction of HRMECs.

Lysophosphatidylcholine (LPC) is the primary component of oxidized low-density lipoprotein (LDL) [14]. The influence of LPC on endothelial cells is crucial in the progression of atherosclerosis and other cardiovascular diseases [15]. LPC can elevate the production of proinflammatory cytokines, such as IL-6, IL-8, and TNF-*α*, which aggregates inflammation and then promotes the development of diseases [16]. Furthermore, LPC results in endothelial cell dysfunction by producing reactive oxygen species (ROS) in the vascular endothelium and induces oxidative stress by elevating the concentration of free Ca^2+^ in the cytoplasm of muscle cells, macrophages, and leukocytes [17]. Plasma LPC concentrations were found significantly elevated in DR [18]. It is demonstrated that increased LPC levels lead to postprandial hyperglycemia by suppressing glucose uptake by muscle, heart, and liver tissues [18]. Hence, our study is aimed at investigating the specific regulatory mechanism of LPC on HG-treated HRMECs.

Toll-like receptors (TLR) are pattern recognition receptors that modulate inflammatory responses when specific molecular patterns found on endogenous damage-related molecules and foreign organisms are detected [19]. TLR4 belongs to the TLR family, which activates the innate immune system [20]. Increasing evidence demonstrated that TLR4-mediated inflammation was associated with diabetic vascular complication and retinopathy [21]. High glucose facilitates the expression of TLR4 and then the activation of TLR4 mediates oxidative stress, aggravating the HRMEC dysfunction and insulin resistance [22]. Additionally, the expression of NF-*κ*B, a downstream factor of TLR4, is significantly elevated in HG-exposed cells, which results in the secretion of inflammatory cytokines [23]. In the previous study, LPC was demonstrated to trigger TLR4-mediated signaling pathway [24]. Therefore, we explored whether LPC regulated the TLR4/NF-*κ*B pathway in HRMECs.

In this study, we aimed to evaluate the influence of LPC on HG-treated HRMECs, which were cocultured with BMSCs. Our findings might provide novel sight into the therapeutic approaches for DR treatment.

## 2. Materials and Methods

### 2.1. Coculture of BMSCs and HRMECs

Bone marrow mesenchymal stem cells (BMSCs) and human retinal microvascular endothelial cells (HRMECs) were provided by Ningbo Mingzhou Biotechnology Co., Ltd. (Zhejiang, China). The transwell chambers (0.4 *μ*m; Corning Life Sciences, Corning, NY) were used for cell coculture, assessing the effects of substances secreted or metabolized by BMSCs on HRMECs. HRMECs were put into the upper chamber while BMSCs were incubated in the bottom chamber. After 24 h, HRMECs were harvested for ELISA.

### 2.2. Cell Culture and Treatment

HRMECs were incubated in endothelial cell medium containing 1% penicillin/streptomycin and 10% fetal bovine serum (FBS; Invitrogen, Carlsbad, CA, USA) at 37°C in a humidified atmosphere with 5% CO_2_. For the glucose treatment, cells were incubated with glucose (Sigma-Aldrich, St. Louis, MO, USA) at a concentration of 25 mM (HG) and 5 mM (NG) for three days after they reached 90% confluence. For LPC treatment, 5 × 10^5^ HRMECs were seeded onto 6-well culture plates. After 24 h, culture medium was changed and various amounts of LPC (10-50 *μ*g/ml; Sigma-Aldrich) were added. After incubation for another three days, medium was harvested for ELISA.

### 2.3. BMSC Isolation, Culture, and Identification

Human BMSCs were incubated in the DMEM-F12 supplement with 10% FBS, L-glutamine, and penicillin-streptomycin solution (both 100x, diluted to 1x for use). The fresh medium was applied to wash off the nonadherent cells after 48 h incubation. The cells of passage 3 with 80% confluence were chosen for BMSC identification.

Human BMSCs were centrifuged after 0.25% trypsin detaching. The precipitates obtained from centrifugation were washed twice with 1x PBS to count the cells. Human BMSCs (1 × 10^6^ cells/ml) were probed with CD45 antibody (ab27287, Abcam, Cambridge, MA, USA), CD105 antibody (ab155367, Abcam), and CD73 antibody (ab157335, Abcam) and subsequently analyzed using flow cytometry.

### 2.4. Enzyme-Linked Immunosorbent Assay (ELISA)

The contents of inflammatory cytokines including interleukin- (IL-) 6, IL-8, and tumor necrosis factor-*α* (TNF-*α*) in HRMECs were assessed according to the procedure of the Simple Step ELISA® kits (Abcam). HRMECs were lysed by RIPA lysis buffer and centrifuged to obtain the supernatant. The content of glutathione (GSH) and the activities of catalase (CAT) and superoxide dismutase (SOD) in the supernatants of cells were examined with glutathione detection kit (A006-2, Nanjing Jiancheng Bioengineering Institute, Nanjing, China), human catalase ELISA kit (ab277396, Abcam), and human superoxide dismutase 1 ELISA kit (ab119520, Abcam) according to the manufacturer's protocols. A microplate reader (Thermo Fisher Scientific) was applied to measure the optical density at 450 nm.

### 2.5. Flow Cytometry

The apoptosis of treated HRMECs was evaluated by Annexin V/fluorescein isothiocyanate (FITC) and propidium iodide (PI) apoptosis detection kits (BD Biosciences, San Jose, CA, USA). Cells were harvested and resuspended in 100 *μ*l of binding buffer at a density of 1 × 10^6^ cells/ml. Then, the cells were double stained with 5 *μ*l of PI and 10 *μ*l of FITC-Annexin V under darkness. Finally, cell apoptotic rate was measured with a FACSCalibur flow cytometry (BD Biosciences).

### 2.6. Western Blotting

HRMECs were harvested and lysed in radio-immunoprecipitation assay (RIPA) lysis buffer (Beyotime, Shanghai, China). Proteins were extracted using a protein extraction kit (Thermo Fisher Scientific). The protein concentration was determined using the BCA protein assay kit (Boster Biological Technology, Wuhan, China). Equal amounts of proteins were then subjected to 10% sodium dodecyl sulfate polyacrylamide gel electrophoresis (SDS-PAGE) and transferred to PVDF membranes (Pall Company, New York, USA). The membrane blocked with 5% nonfat milk powder was then incubated at 4°C overnight with the primary antibodies against TLR4 (ab13556, 1 : 500, Abcam), p-NF-*κ*B (ab194908, 1 : 1000, Abcam), and *β*-actin (ab8227, 1 : 1000, Abcam, Cambridge, MA, USA). Subsequently, the membranes were washed and incubated with horseradish-peroxidase-conjugated secondary antibody at room temperature for 2 h. The protein bands were detected using an enhanced chemiluminescence reagent (ECL) kit (Pierce, Rockford, IL, USA). The data was normalized to *β*-actin as an internal control.

### 2.7. Statistical Analysis

The data were analyzed using GraphPad Prism 6.0 software (GraphPad Software, San Diego, CA, USA). The data were presented as the mean ± standard deviation (SD). The comparisons between two or more groups were assessed using Student's *t-*test or one-way analysis of variance (ANOVA) followed by Tukey's post hoc test. Statistical significance compared to the controls was denoted by ^∗^*p* < 0.05, ^∗∗^*p* < 0.01, and ^∗∗∗^*p* < 0.001.

## 3. Results

### 3.1. BMSC Identification

**Figure 1:**
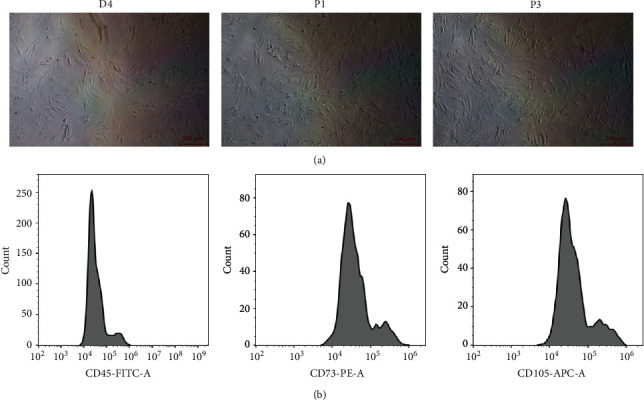
BMSC identification.

The number of BMSCs was found to be increased followed by cell colony fusion on the 4^th^ day using a light microscope. There existed no shape difference between BMSCs of passages 1 and 3, both of which manifesting long fusiform (Figure 1(a)). In addition, BMSC identification demonstrated that CD105 and CD73 were positively expressed while CD45 was negatively expressed in BMSCs of passage 3 (Figure 1(b)).

### 3.2. BMSCs Alleviate Inflammatory Response of HG-Treated HRMECs

**Figure 2:**
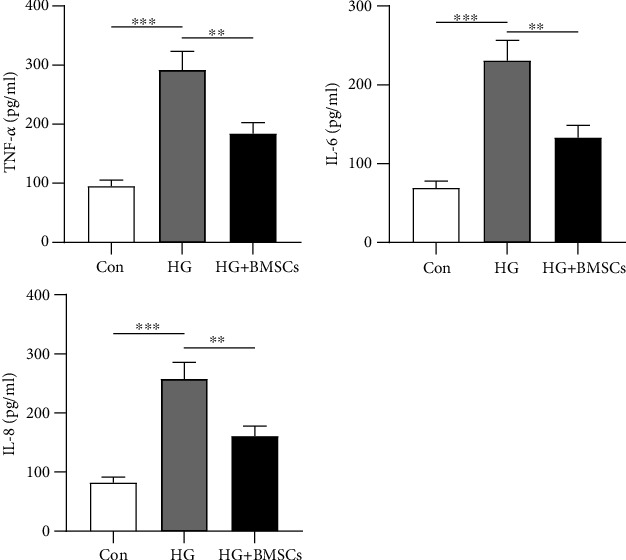
BMSCs alleviate inflammatory response of HG-treated HRMECs. (a) ELISA was carried out to evaluate the content of inflammation factors IL-8, IL-6, and TNF-*α* in HG-treated HRMECs after BMSC coculture. ^∗∗^*p* < 0.01 and ^∗∗∗^*p* < 0.001.

The levels of inflammatory cytokines such as IL-8, IL-6, and TNF-*α* were found upregulated in DR patients and might have a synergistic effect on the pathogenesis of DR [25]. In HG-treated HRMECs, IL-8, IL-6, and TNF-*α* contents were elevated (2-folds, ^∗∗∗^*p* < 0.001), while BMSCs reversed (40% reduction, ^∗∗^*p* < 0.01) the increased levels of TNF-*α*, IL-6, and IL-8 induced by HG (Figure 2(a)). Therefore, BMSCs hindered inflammatory response of HG-treated HRMECs.

### 3.3. BMSCs Alleviate Oxidative Stress Injury and Apoptosis in HG-Treated HRMECs

**Figure 3:**
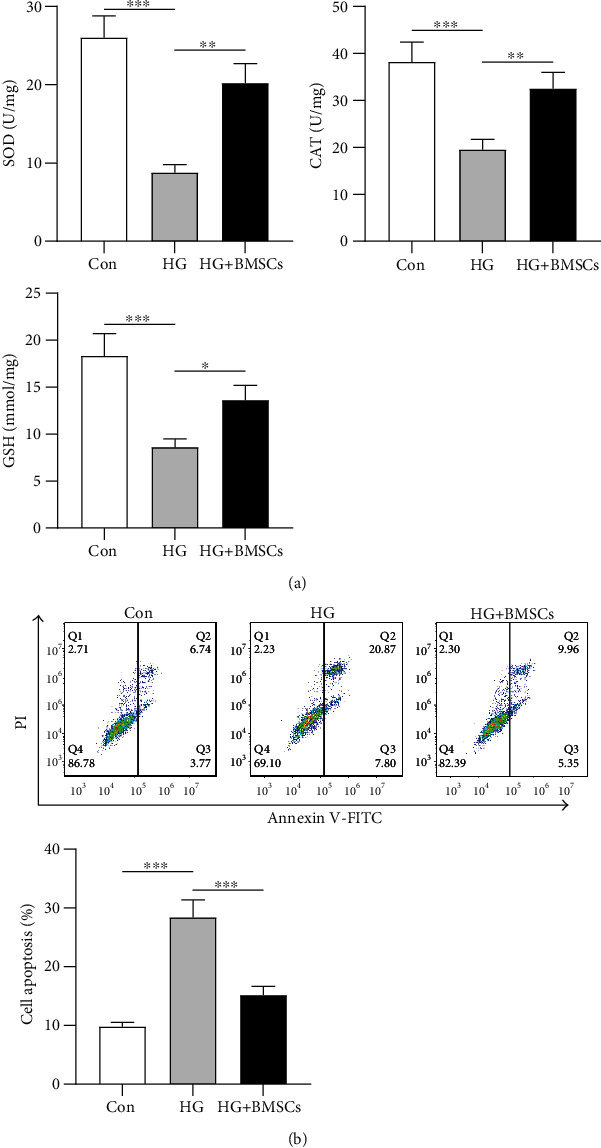
BMSCs alleviate oxidative stress injury and apoptosis in HG-treated HRMECs. (a) ELISA was conducted to detect CAT and SOD activities and GSH content in HG-treated HRMECs after BMSC treatment. (b) Flow cytometry analysis was performed to probe HG-treated HRMEC apoptosis after coculture with BMSCs. ^∗∗^*p* < 0.01 and ^∗∗∗^*p* < 0.001.

SOD, CAT, and GSH are antioxidant-related enzymes and molecules of oxidative stress injury [26]. The levels of GSH, CAT, and SOD were reported suppressed in HG-induced DR [27]. In our study, CAT and SOD activities and GSH content were reduced (49%-66% reduction, ^∗∗∗^*p* < 0.001) in HG-treated HRMECs, and the decrease mediated by HG was rescued (1.5- to 2.3-folds, ^∗∗^*p* < 0.01) by BMSC treatment (Figure 3(a)). Flow cytometry indicated that the enhancement of HRMEC apoptosis was observed in the HG group. However, coculture of BMSCs and HRMECs reversed (47% reduction, ^∗∗∗^*p* < 0.001) the promoting influence (3-folds, ^∗∗∗^*p* < 0.001) of HG on cell apoptosis (Figure 3(b)). It can be summarized that BMSCs attenuated oxidative stress injury and cell apoptosis in HG-treated HRMECs.

### 3.4. LPC Offsets the Protective Effect of BMSCs on Inflammatory Response of HRMECs

**Figure 4:**
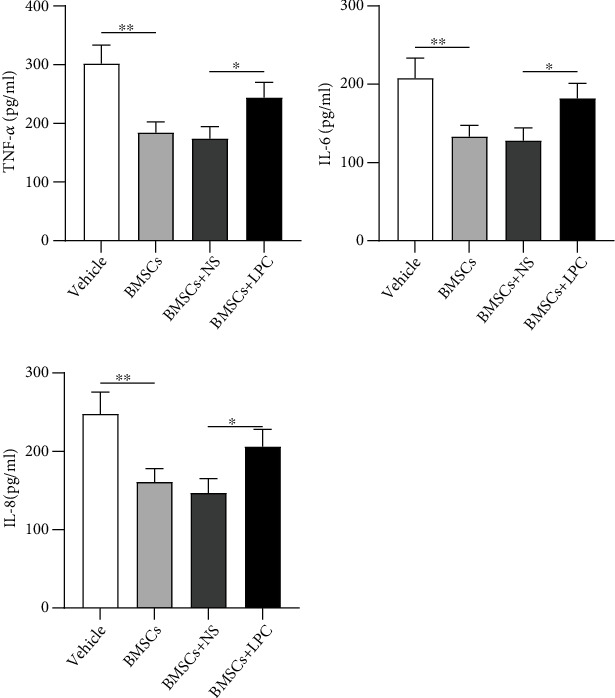
LPC offsets the protective effects of BMSCs on inflammatory response of HRMECs. (a) ELISA was performed to assess the content of inflammation factors IL-8, IL-6, and TNF-*α* in BMSC-cocultured HRMECs after LPC treatment. ^∗^*p* < 0.05 and ^∗∗^*p* < 0.01.

LPC is a potent inflammatory lipid, and increased levels of LPC are associated with the onset of DR [18]. We further investigated whether LPC influenced the protective effect of BMSCs on HRMECs in response to HG. The levels of IL-8, IL-6, and TNF-*α* were significantly reduced (35-39% reduction, ^∗∗^*p* < 0.01) in HRMECs after coculture with BMSCs. Moreover, we found that the inhibitory effects of BMSCs on the levels of inflammation factors were partially reversed (40%-42% increase, ^∗^*p* < 0.05) by LPC treatment (Figure 4(a)). These data suggested that LPC offsets the protective effect of BMSCs on inflammatory response of HRMECs.

### 3.5. LPC Offsets the Protective Effect of BMSCs on Oxidative Stress Injury of HRMECs

**Figure 5:**
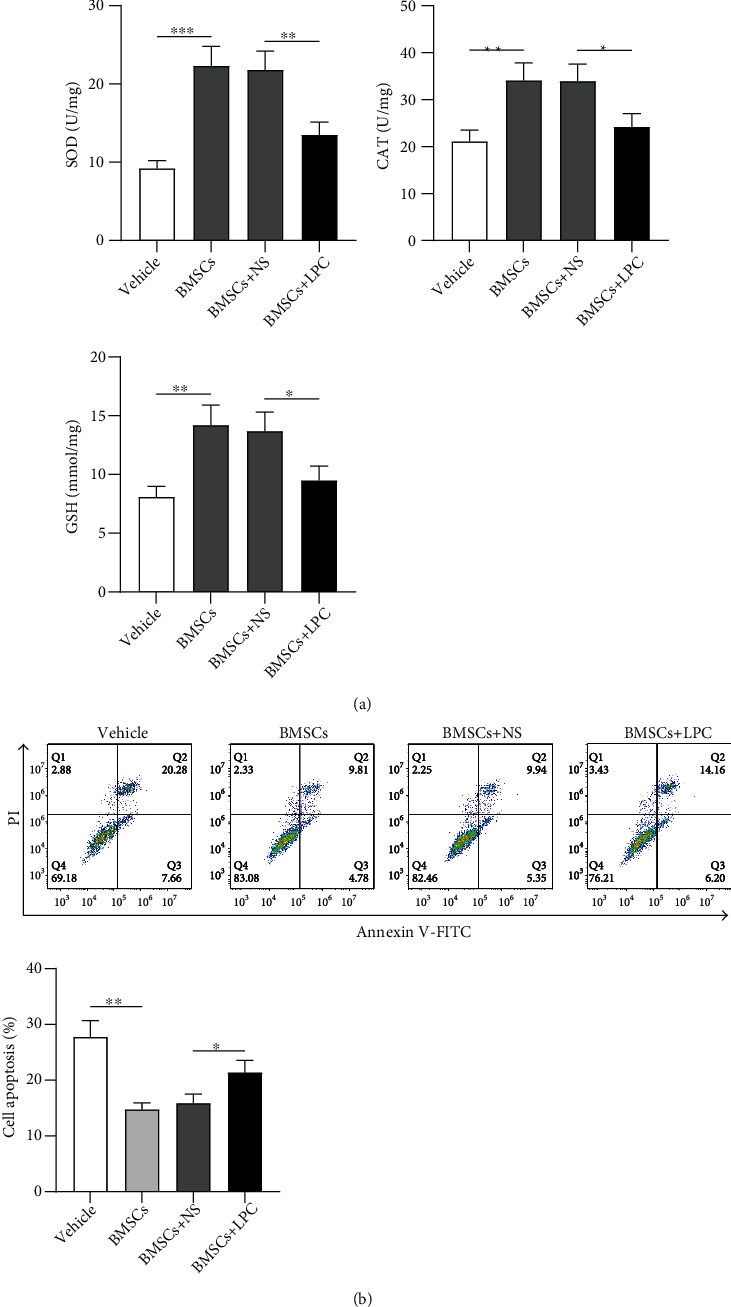
LPC offsets the protective effects of BMSCs on oxidative stress injury of HRMECs. (a) The activities of CAT and SOD and the content of GSH in BMSC-cocultured HRMECs after LPC treatment were subjected to ELISA. (b) BMSC-cocultured endothelial cell apoptosis after LPC treatment was examined by flow cytometry analysis. ^∗^*p* < 0.05, ^∗∗^*p* < 0.01, and ^∗∗∗^*p* < 0.001.

Next, we evaluated the influence of LPC on levels of oxidative stress-related markers. CAT and SOD activities and GSH content were elevated (1.1- to 2.4-folds, ^∗∗∗^*p* < 0.001 and ^∗∗^*p* < 0.01) in HRMECs after being cocultured with BMSCs. However, LPC treatment partially abolished (29%-38% inhibition, ^∗^*p* < 0.05 and ^∗∗^*p* < 0.01) the protective effect of BMSCs on oxidative stress injury of HRMECs (Figure 5(a)). Additionally, flow cytometry analysis also demonstrated that HRMEC apoptosis inhibited (47% decrease, ^∗∗^*p* < 0.01) by BMSC treatment was reversely enhanced (35% improvement, ^∗^*p* < 0.05) by LPC treatment (Figure 5(b)). In conclusion, LPC offsets the protective effect of BMSCs on oxidative stress injury of HRMECs and abolished the inhibitory influence of BMSCs on cell apoptosis.

### 3.6. LPC Activates the TLR4/NF-*κ*B Signaling Pathway

**Figure 6:**
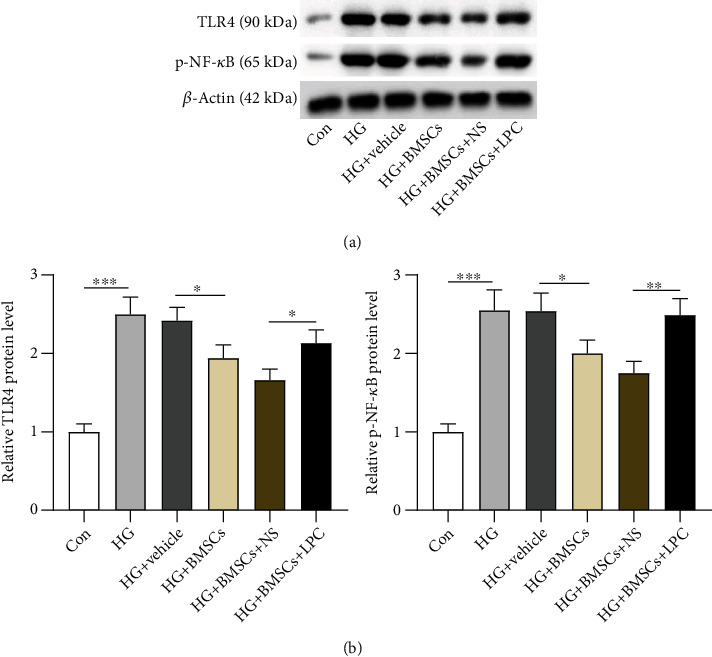
LPC activates the TLR4/NF-*κ*B signaling pathway. (a, b) The levels of TLR4 and p-NF-*κ*B proteins in HG-treated HRMECs after BMSC coculture or LPC treatment were examined by western blotting. ^∗^*p* < 0.05, ^∗∗^*p* < 0.01, and ^∗∗∗^*p* < 0.001.

LPC was previously reported to regulate the level of TLR4 and NF-*κ*B p65 proteins [28]. We then detected the protein levels of TLR4 and phosphorylated NF-*κ*B (p-NF-*κ*B). In HG-treated HRMECs, the levels of TLP4 and p-NF-*κ*B proteins were upregulated (2.5-folds, ^∗∗∗^*p* < 0.001), and their protein levels were downregulated (20% decrease, ^∗^*p* < 0.05) in HG-treated HRMECs cocultured with BMSCs. Moreover, LPC treatment partially reversed (28% and 42% increase, ^∗^*p* < 0.05 and ^∗∗^*p* < 0.01) the downregulated protein levels induced by BMSCs (Figures 6(a) and 6(b)). Therefore, we concluded that LPC activated the TLR4/NF-*κ*B signaling pathway.

## 4. Discussion

DR, as a common and specific microvascular complication of diabetes, remains a main cause of preventable blindness among working-aged people [29]. The inflammation and oxidative stress of endothelial cells are the indicatives of DR [30]. In serum and ocular samples collected from diabetic patients with DR, many inflammatory cytokines and chemokines are found to be increased [31]. In addition, oxidative stress is regarded as the main target in the pathophysiology of diabetic retinopathy [32]. Previous studies have indicated that BMSCs can alleviate the dysfunction of endothelial cells and in turn relieve the development of DR [33]. Clinically, BMSC was used to treat a 77-year-old male patient with visual loss [34]. We herein initially cocultured HG-treated HRMECs with BMSCs, and we discovered that the oxidative stress and inflammation of HRMECs were mitigated by BMSC treatment.

As a significant lysophospholipid, LPC can elevate levels of blood glucose in diabetic mice and alleviate inflammation [35]. LPC was found to be significantly elevated in plasma of diabetic patients [36], and total serum LPC was higher in adults with neovascular age-related macular degeneration [37]. LPC is the major enzymatic product of lipoprotein-associated phospholipase A2 (Lp-PLA2) [38]. The previous study investigated the effects of LPC and Lp-PLA2 on diabetes-induced retinal vasopermeability via animal experiments [39]. The results showed that LPC and Lp-PLA2 participated in blood-retinal barrier (BRB) damage during DR. In diabetic rats, Lp-PLA2 inhibition was demonstrated to effectively suppress BRB breakdown and protect against BRB dysfunction via LPC, which was shown to induce vascular permeability to retinal vascular endothelium through VEGF receptor 2 (VEGFR2) [39]. However, the previous study only explored the function of LPC in diabetic animal models, while whether LPC played a role during DR development of DR patients remained further elucidation. Therefore, in our study, we treated HRMECs with LPC and detected the influence of LPC on the dysfunction of HRMECs. Previously, mouse BMSC-derived exosomes were demonstrated to protect against DR [33], and intravitreal BMSCs were demonstrated to have the potential to improve visual function through experiments on diabetic rats [40]. Then, our present study first explored the influence of human BMSCs on inflammatory response and oxidative stress in DR development. The results indicated that human BMSCs alleviated inflammatory response and oxidative stress injury of HG-treated HRMECs. However, LPC offsets the protective effects of BMSCs on the inflammatory response and oxidative stress injury of HRMECs.

LPC was reported to act as a multiactivity ligand, binding to various proteins including TLR4, triggering a series of proinflammatory and prooxidant pathways [41]. TLR4 predisposes DR, and its overexpression in endothelial cells results in the enhanced inflammatory responses and even the pathogenesis of DR [42]. It has been demonstrated in many studies that the TLR4/NF-*κ*B axis inhibition alleviated the onset and progression of several diabetes concomitant diseases. For example, GSDMD expression was elevated in diabetic kidney disease, while suppressing the TLR4/NF-*κ*B pathway induced GSDMD-related proptosis in DKD [43]. Berberine ameliorated diabetic nephropathy by relieving inflammatory response and STZ-induced renal injury through inactivating the TLR4/NF-*κ*B pathway [44]. The previous study concluded that BMSC-derived exosome protected against DR via repressing the TLR4/NF-*κ*B signaling pathway [33], indicating that the TLR4/NF-*κ*B signaling pathway exerts definite effects on DR development. In our study, we evaluated the level of TLR4 and p-NF-*κ*B protein after LPC treatment to explore whether LPC can activate the TLR4/NF-*κ*B pathway. We found that LPC offsets the protective effects of BMSCs on inflammatory response and oxidative stress injury of HRMECs via TLR4/NF-*κ*B signaling. However, the findings of our study remained to be perfected through further *in vivo* experiments to verify the influence and underlying mechanism of LPC in DR treatment.

In summary, LPC offsets the protective effects of BMSCs on inflammatory response and oxidative stress injury of HRMECs via the TLR4/NF-*κ*B signaling. This study might widen our mind to DR treatment with LPC/TLR4/NF-*κ*B-based targeted therapy in the future.

## Data Availability

The data used to support the findings of this study are available from the corresponding author upon request.
